# Perception, confidence, and willingness to respond to in-flight medical emergencies among medical students: a cross sectional study

**DOI:** 10.1080/07853890.2024.2337725

**Published:** 2024-04-08

**Authors:** Majed Alnabulsi, Ehab Abdelhalim Abo Ali, Mohammad Hassan Alsharif, Najla’a Fathi Filfilan, Sahar Hamed Fadda

**Affiliations:** a Department of Internal Medicine, Medicine Program, Batterjee Medical College, Jeddah, Saudi Arabia; b Department of Community Medicine, Medicine Program, Batterjee Medical College, Jeddah, Saudi Arabia; c Public Health and Community Medicine Department, Faculty of Medicine, University of Tanta, Tanta, Egypt; d Medicine Program, Batterjee Medical College, Jeddah, Saudi Arabia

**Keywords:** Medical education, emergency medicine, first responder, aviation medicine

## Abstract

**Background:**

In-flight medical emergencies (IMEs) are expected to increase as air travel normalized in the post-COVID-19 era. However, few studies have examined health professions students’ preparedness to respond to such emergencies. Therefore, this study aimed to investigate medical students’ knowledge, confidence, and willingness to assist during an IME in their internship program.

**Methods:**

This cross-sectional survey utilized an online, self-administered questionnaire-based survey targeted at medical students at two medical colleges in Saudi Arabia. The questionnaire comprised three parts: sociodemographic characteristics, knowledge about aviation medicine (10 items), and confidence (7 items)/willingness (4 items) to assist during an IME. Odds Ratios (OR) and 95% Confidence Intervals (95%CI) were computed to detect potential associations between the knowledge levels and the other independent variables. Responses to confidence and willingness questions were scored on a 5-point Likert scale.

**Results:**

Overall, 61.4% of participants had inadequate knowledge scores for providing care during an IME, and the proportion of participants did not differ between those who had or had not attended life support courses (60.4% vs. 66.7%, *p* > 0.99). Only frequency of air travel ≥ two times per year was associated with higher odds of adequate knowledge score [OR = 1.89 (95%CI 1.14–3.17), *p* = 0.02]. In addition, 93.3% of the participants had low, 6.3% had moderate, and 0.8% had high willingness scores, while 86.3% had low, 12.2% had moderate, and 1.5% had high confidence scores. There were no differences in the proportion of participants with low, moderate, and high willingness or confidence scores by attendance in life support courses.

**Conclusion:**

Even though over 8 in 10 students in our study had previously attended life support courses, the overwhelming majority lacked the knowledge, confidence, and willingness to assist. Our study underscores the importance of teaching medical students about IMEs and their unique challenges before entering their 7th-year mandatory general internship.

## Introduction

The number of passengers using air transport worldwide doubled from 2.25 billion in 2009 to 4.46 billion in 2019, with an average annual growth rate of 7.1% in the decade before the COVID-19 pandemic [1]. The increasing number of air passengers necessitates better preparedness to handle in-flight medical emergencies (IME). The global incidence of IME is estimated at 18.2 events per million passengers, with an all-cause mortality rate of 0.21 per million passengers [2]. As the aviation industry recovers from the 60% decrease in air passengers in 2020 due to the COVID-19 pandemic [1], which the International Air Transport Association (IATA) projects to exceed pre-COVID-19 levels in 2024 [3], the number of IMEs will likely increase as well [4,5].

Most IMEs are minor events such as dizziness, headaches, syncope, nausea or vomiting, shortness of breath, and injuries/burns [6,7]. Although airline cabin crew are trained to handle IMEs, recent reports indicate that only a third of IMEs are handled by the cabin crew without the assistance of an onboard health professional [6,7]. In one study, physicians were the most frequent (48.1%) volunteers responding to the cabin crew’s request for help [4]. Nurses, emergency medical service providers, and other healthcare professionals accounted for 28.2% of volunteers [4]. Given the physiological impacts of air travel on the human body and the logistical challenges of limited space and resources, IMEs present a unique challenge to even the most experienced healthcare providers [8–12]. Additionally, for many physicians, the medicolegal consequences may be a barrier to assisting during an IME [13].

As future healthcare providers, medical students must be prepared to handle these emergencies, as they may encounter them during their careers. For example, Katzer et al. [14] showed that about one in five senior medical students had experienced a medical emergency while flying. Given that in-flight physiology, epidemiology, medicolegal implications, and available resources for healthcare respondents are not routinely taught in medical curricula, it is not surprising that IMEs can be particularly daunting to medical students as they lack both confidence and competence to provide adequate medical care in such settings [14]. In a previous study, only 11.5% of primary care doctors exhibited confidence in providing medical assistance during an IME [11].

While only a limited number of studies have investigated the competency of medical students at various levels, their findings consistently indicate that these students feel inadequately prepared to respond to in-flight medical emergencies and may possess sub-optimal knowledge in this area [13,14]. Therefore, this study evaluated medical students’ knowledge, confidence, and willingness to respond to IMEs in two large medical colleges in the Western region of Saudi Arabia.

## Materials and methods

### Conceptual framework

The Saudi Medical Education Directives (SaudiMED) framework was launched in 2009 by the Deanery of Saudi Medical Colleges in line with the global trend in competency-based medical education [15]. The framework identifies the key clinical conceptual, procedural, and professional competencies that medical students should achieve by the start of their mandatory general internship in the 7th year [15,16]. Although the SaudiMED framework covers common clinical presentations during an IME, such as syncope, lightheadedness, abnormal breathing, chest pain, arrhythmias, and abdominal pain [5], and necessary skills to manage an IME such as performing patient evaluation, first-aid, basic cardiac life support, and basic burn care, it does not include these competencies in the context of IMEs which may require additional considerations and understanding of role of medical volunteers and flight crew during an IME.

For instance, Ho et al. [17] identified the need for doctors and medical students first to assess their competencies and physical/mental state for providing inflight care. If competent, the doctor has the ethical obligation to identify themselves and communicate their competencies to the flight crew, after which the duty of care and legal obligations are established. Medical students should explicitly explain to the flight crew that they can only assist in a limited capacity. While assisting, doctors and medical students should communicate and collaborate with the flight crew, obtain the patient’s consent, and keep a detailed clinical record. The doctor’s role during an IME is to assist the pilot and the crew, and the ultimate decision for flight diversion rests with the pilot. Medical volunteers should be aware of the inflight environment and how it affects bodily processes, the resources available in onboard medical kits, and if the pilot decides to divert the flight, then assist the pilot in establishing clear and thorough communication with the ground crew for adequate preparedness on arrival. In addition, Hu and Smith [18] recommend that the medical volunteer understand that the personal legal risk is small unless negligent and should provide medical aid confidently.

We designed a multicentered survey to investigate if the clinical competencies envisioned by the SaudiMED framework sufficiently empower medical students by the start of their internship programs with the necessary knowledge, confidence, and willingness to assist during an IME while considering the role and expertise of medical volunteers required during an IME as outlined by Ho et al. [17] and Hu and Smith [18].

### Study setting

In Saudi Arabia, the higher education landscape has traditionally been dominated by public universities and colleges, typically with one major university per city. However, various private colleges have been established recently, leading to numerous educational institutions in Jeddah. For this study, we specifically selected two institutions: King Abdulaziz University, the only public medical school in Jeddah, and Batterjee Medical College, the largest private college in the region, where the authors of this paper primarily work.

### Study population

The study targeted medical students doing their 7th-year mandatory general internship, a prerequisite for graduation from medical school in Saudi Arabia. A convenience sampling approach was used for data collection.

### Survey method

This multicenter cross-sectional survey was conducted from June 2022 to December 2022 using an online, self-administered questionnaire-based survey. The questionnaire consisted of three sections, covering sociodemographic characteristics (eight items), knowledge about aviation medicine (ten items), and confidence/willingness to assist during an IME (assessed through seven items and four items, respectively).

The questionnaire was developed after an extensive literature review. Various components from previously published work [11,13,14] in this domain were adapted into our questionnaire, which comprised three main sections. The first section covered sociodemographic parameters such as age, gender, type of university, air travel frequency, completing basic life support (BLS) or advanced cardiac life support (ACLS) courses, previous encounters with IMEs, and completing the emergency medicine rotation. The second part contained ten questions (two multiple-choice and eight true/false) designed to test the knowledge in four domains: aviation pathophysiology (three items), epidemiology (two items), equipment/support availability (two items), and medicolegal responsibilities (three items). The final section included questions to test students’ confidence (seven statements) and their willingness (four statements) to provide medical care during an IME (e.g. ‘My medical education has given me sufficient knowledge and skill to provide medical assistance during a medical emergency’ and ‘I would identify myself as a medical intern and offer assistance in the event of an in-flight medical emergency’).

### Pilot

Three independent physician experts with backgrounds in emergency medicine and academic medicine (see acknowledgments for details) were involved in developing the survey instrument to assess the content and face validity of the questionnaire. Subsequently, twenty students who were not a part of the final study sample were recruited for pilot testing. The sample size for the pilot test was based on the study by Hertzog [19], which demonstrated that 20 participants per group are adequate in pilot tests.

Cronbach’s alpha [20] was used to test the internal consistency for confidence (α = 0.899) and willingness (α = 0.721) item scores in the pilot test. Generally, Cronbach’s alpha >0.7 is considered the minimal threshold for acceptable internal consistency, with a very high value (>0.9) suggestive of redundancy in the questionnaire [21]. Hence, we concluded that our survey instrument had acceptable internal consistency and low redundancies, and no significant changes were made to the questionnaire for the main survey.

### Data collection

The survey was developed using the Google Forms survey platform, and an online link to the survey was shared with students at the two participating medical colleges. We distributed the survey link through various channels, including students’ WhatsApp groups and email accounts to ensure maximum participation. The link did not permit multiple responses from the same participant to prevent duplicate responses.

### Data analysis

The outcome variables were levels of knowledge, confidence, and willingness to volunteer during an IME. Qualitative variables were presented as numbers and frequencies, and quantitative variables were presented as mean ± SD.

The percentage of correct responses to the ten questions in the knowledge section was ranked from highest = 1 to lowest = 10. Overall domain knowledge was calculated by taking the average of correct responses to individual questions within the domain. In the subgroup analysis, Odds Ratios (OR) and 95% Confidence Intervals (95%CI) were computed to compare the proportion of correct responses on each of the ten questions by participants who had or had not completed BLS or ACLS courses to assess if the participation was associated with answering the knowledge-related questions correctly. Statistical significance was assessed using Fisher’s exact test with a *p-*value of <0.05 considered statistically significant.

In addition, OR and 95%CI were computed to detect potential associations between the knowledge levels and the other independent variables. For this analysis, knowledge level was dichotomized as ‘adequate’ or ‘inadequate.’ Achieving ≥ 60% of correct answers in the knowledge domain was considered ‘adequate,’ mirroring the usual acceptable pass rate in academia.

Questions related to confidence and willingness were assessed using a 5-point Likert-type scale, ranging from ‘strongly disagree’ (scored as 0) to ‘strongly agree’ (scored as 4). The total willingness and confidence scores were derived by adding the score of the responses with a maximum achievable score of 28 for confidence and 16 for willingness domains. A total score of <70% was categorized as ‘low’ confidence/willingness to assist during an IME, a score between ≥70% and 89% was categorized as ‘moderate’ confidence/willingness, and a score ≥90% was categorized as ‘high’ confidence/willingness in line with previous studies [13,22]. In the subgroup analysis of students who had or had not completed BLS or ACLS courses, the proportion of students achieving low, moderate, or high confidence/willingness was assessed using 2-way ANOVA. A *p*-value of >0.05 was considered statistically significant. For comparison, Cronbach’s alpha was used to test the internal consistency of confidence and willingness item scores obtained during the main survey with a threshold similar to that used in the pilot study.

All statistical analysis was conducted using Statistical Package for Social Sciences (SPSS) Software (Version 17.0, SPSS, Inc, Chicago, IL, USA).

### Ethics

Ethical approval was granted by the Institutional Review Board (IRB) of the research unit at Batterjee Medical College, Jeddah, Saudi Arabia (reference number RES-2022-0028). Written informed consent was prompted as the first question in the survey and was obtained from all participants who wished to complete the survey in compliance with our institutional approval. Confidentiality of the data was guaranteed.

## Results

A total of 117 male and 277 female medical students with a median age of 23 years (IQR 22–24) responded to our questionnaire. Over one-third of the participants attended a public medical university, and 81% had a grade point average of 3.75 to 5.0. More than half the participants (53.2%) stated that they travel at least once per year, and 14.2% had encountered at least one IME. While 84% of the participants had attended life support courses like BLS or ACLS, only 40.4% had completed the emergency medicine rotation. The study participants’ detailed demographic and academic characteristics are presented in Table 1.

**Table 1.** Demographic and academic characteristics of medical students (*n* = 394).

Variables		*n*	*%*
**Gender**	*Female*	277	70.3
	*Male*	117	29.7
**Type of medical school**	*Public*	261	66.2
	*Private*	133	33.8
**GPA**	*2.00 − 2.74*	6	1.5
	*2.75 − 3.74*	69	17.5
	*3.75 − 4.49*	160	40.6
	*4.5 − 5.00*	159	40.4
**Frequency of air travel**	*<1 time per year*	184	46.7
	*One time per year*	105	26.6
	*≥ 2 times per year*	105	26.6
**Having encountered an IME**	*Yes*	56	14.2
	*No*	338	85.8
**Having attended life support courses**	*Yes*	331	84.0
	*No*	63	16.0
**Having completed the emergency medicine rotation**	*Yes*	159	40.4
	*No*	235	59.6

### Knowledge of providing medical care during an IME

The percentage of participants with correct responses for each question is shown in Table 2. When analyzed by domain, 77% of the participants answered questions about equipment availability/support systems correctly, followed by questions about aviation pathophysiology (53.6%), medicolegal responsibilities (39.9%), and epidemiology of IMEs (35.8%) (Table 2). In the subgroup analysis, attending life support courses was associated with higher odds of answering just one question correctly [Q10: Medical assistance rendered by a capable physician is of little personal legal risk and is supported by experts in aviation medicine. OR = 1.8 (95%CI 1.04–3.1), *p* = 0.04; Table 2].

**Table 2.** Knowledge about in-flight medical emergencies of medical students (*n* = 394).

	Overall				Life support training				No life support training				OR (95%CI)	*p*
	n	%_CR_	Rank	%_DCR_	n	%_CR_	Rank	%_DCR_	n	%_CR_	Rank	%_DCR_		
**Aviation Pathophysiology**														
Q1: Commercial airplane cabins are typically pressurized to an altitude of sea level	97	24.6	9	53.6	80	24.2	9	53.7	17	5.1	8	10.2	0.86	0.63
													(0.48–1.57)	
Q2: The aircraft cabin has a lower partial pressure of oxygen at altitude, with resultant mild hypoxia in healthy passengers	278	70.6	2		234	70.7	2		44	13.3	2		1.04	0.88
													(0.57–1.88)	
Q3: Gas in body cavities can expand by 30% at low cabin pressure associated with cruising attitudes.	259	65.7	4		219	66.2	5		40	12.1	3		1.12	0.67
													(0.63–1.95)	
**Epidemiology**														
Q4: The most common medical emergency occurring during a flight	91	23.1	10	35.8	78	23.6	10	35.4	13	3.9	10	7.3	1.19	0.74
													(0.62–2.34)	
Q5: Majority of in-flight medical emergencies end up requiring diversion of the airplane to the nearest medical facility	191	48.5	6		156	47.1	6		35	10.6	5		0.71	0.27
													(0.41–1.21)	
**Equipment availability/Support system**														
Q6: Cabin Crews are trained in basic life support	341	86.5	1	77.0	290	87.6	1	78.0	51	15.4	1	13.8	1.66	0.16
													(0.84–3.36)	
Q7: Most airlines provide in-flight medical consultation services with ground-based physicians in the event of in-flight medical emergencies.	266	67.5	3		226	68.3	3		40	12.1	3		1.24	0.47
													(0.7–2.16)	
**Medicolegal responsibilities**														
Q8: Doctors are legally required to introduce themselves in the case of an inflight medical emergency	102	25.9	8	39.9	85	25.7	8	40.3	17	5.1	8	7.2	0.94	0.88
													(0.5–1.69)	
Q9: The decision to divert the airplane to the nearest hospital in case of an in-flight medical emergency is usually made by which of the following?	117	29.7	7		95	28.7	7		22	6.6	7		0.75	0.37
													(0.43–1.31)	
Q10: Medical assistance rendered by a capable physician is of little personal legal risk and is supported by experts in aviation medicine.	253	64.2	5		220	66.5	4		33	10	6		1.8	0.04
													(1.04–3.1)	

%_CR_= percentage of participants with correct answers to the question. %_DCR_= percentage of correct answers in the domain.

**Figure 1.**
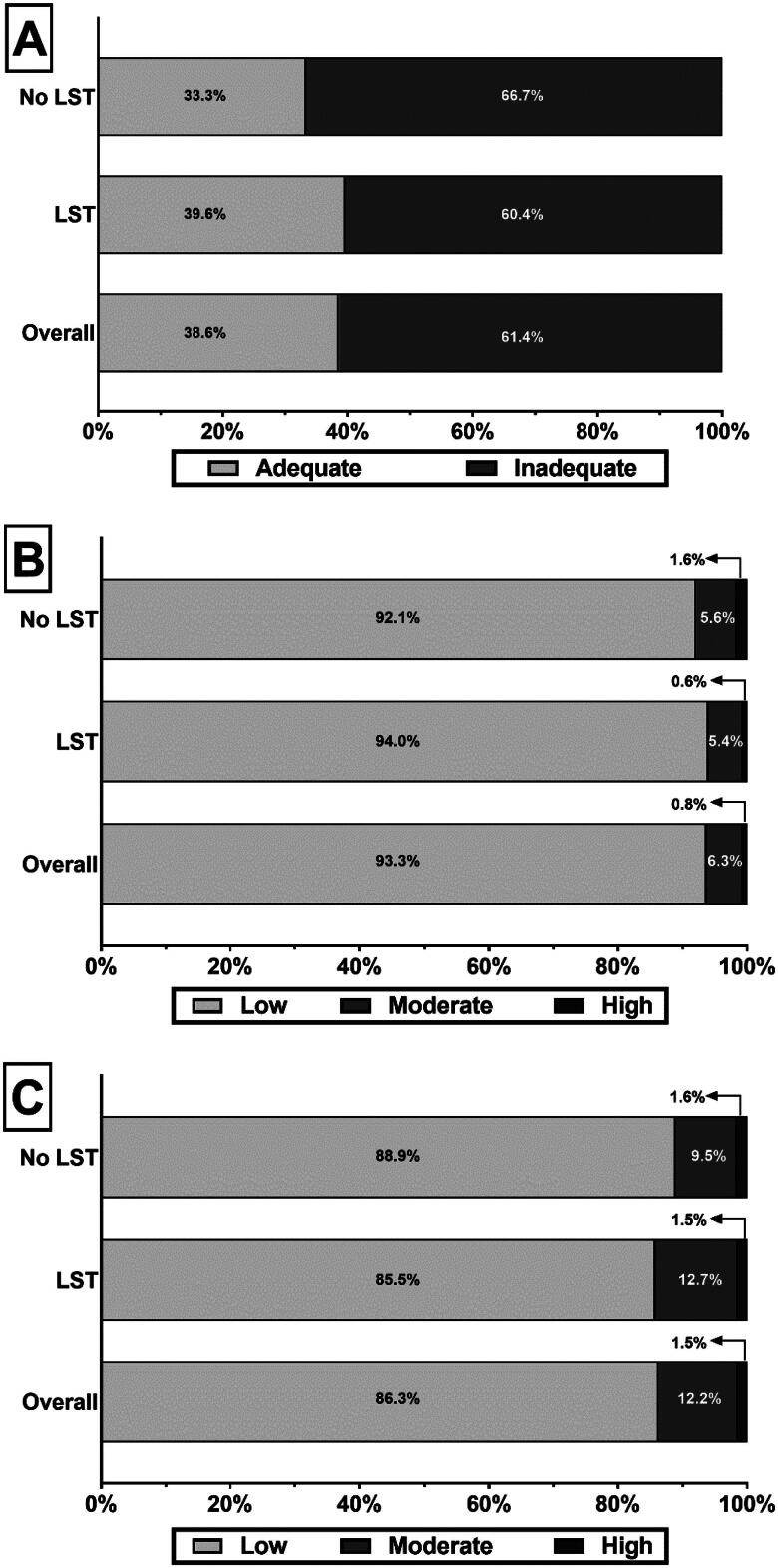
The proportion of survey participants with (A) adequate knowledge, (B) confidence, and (C) willingness to assist during an inflight medical emergency. LST = Life support courses.

However, 61.4% of participants had inadequate knowledge scores for providing care during an IME (*p* = 0.0224; Figure 1A). The proportions of participants with adequate knowledge were numerically higher among those who had attended life support courses than those who had not, but this difference was not statistically significant (39.6% vs. 33.3%, *p* > 0.99; Figure 1A).

Only frequency of air travel ≥ two times per year was associated with higher odds of adequate knowledge score [OR = 1.89 (95%CI 1.14–3.17), *p* = 0.02]; we did not detect any significant association between adequate knowledge level and gender, type of medical school, GPA, age, a past encounter with an IME, attending life support courses, or completing an emergency medicine rotation (Table 3).

**Table 3.** Association between demographic/academic characteristics of medical students and their level of knowledge about in-flight medical emergencies (IME).

Variables		Knowledge level^§^				OR (95%CI)	*p*
		Inadequate (n = 289)		Adequate (n = 171)			
		*n*	*%*	*N*	*%*		
**Gender**	*Male*	76	65	41	35	*(Ref)*	
	*Female*	166	59.9	111	40.1	1.24 (0.8–1.92)	0.37
**Type of medical school**	*Public*	160	61.3	101	38.7	*(Ref)*	
	*Private*	82	61.7	51	38.3	1.02 (0.66–1.55)	>0.99
**GPA**	*2.00 − 2.74*	5	83.3	1	16.7	*(Ref)*	
	*2.75 − 3.74*	42	60.9	27	39.1	0.31 (0.03–2.58)	0.4
	*3.75 − 4.49*	94	58.8	66	41.3	0.28 (0.02–2.17)	0.4
	*4.5 − 5.00*	101	63.5	58	36.5	0.35 (0.03–2.66)	0.42
**Age**	*20*	18	60	12	40	*(Ref)*	
	*21*	33	70.2	14	29.8	1.57 (0.59–4.17)	0.46
	*22*	38	61.3	24	38.7	1.06 (0.44–2.64)	>0.99
	*23*	45	53.6	39	46.4	0.77 (0.35–1.75)	0.67
	*24*	52	63.4	30	36.6	1.16 (0.51–2.69)	0.83
	*25*	24	55.8	19	44.2	0.84 (0.32–2.12)	0.81
	*26*	8	61.5	5	38.5	1.07 (0.27–3.92)	>0.99
	*27*	10	71.4	4	28.6	1.67 (0.44–5.65)	0.52
	*28*	6	75	2	25	2 (0.37–10.85)	0.68
	*29*	2	100	0	0	Inf (0.28-Inf)	0.52
	*30*	2	66.7	1	33.3	1.33 (0.14–20.76)	>0.99
	*>30*	4	66.7	2	33.3	1.33 (0.26–7.81)	>0.10
**Frequency of air travel**	*<1 time per year*	107	58.2	77	41.8	*(Ref)*	
	*One time per year*	59	56.2	46	43.8	0.92 (0.57–1.51)	0.8
	*≥ 2 times per year*	76	72.4	29	27.6	1.89 (1.14–3.17)	0.02
**Having encountered an IME**	*Yes*	32	57.1	24	42.9	*(Ref)*	
	*No*	210	62.1	128	37.9	1.23 (0.69–2.15)	0.55
**Having attended life support courses**	*Yes*	200	60.4	131	39.6	*(Ref)*	
	*No*	42	66.7	21	33.3	1.31 (0.75–2.26)	0.4
**Having completed the emergency medicine rotation**	*Yes*	90	56.6	69	43.4	*(Ref)*	
	*No*	152	64.7	83	35.3	1.4 (0.92–2.14)	0.11

**^§^**Achieving ≥ 60% of correct answers in the knowledge domain was considered "adequate," mirroring the usual acceptable pass rate in academia.

### Confidence and willingness to provide medical care during an IME

Cronbach’s alpha values for the confidence and willingness item scores in the main survey were 0.91 and 0.74, respectively, comparable to the pilot study, indicating good reliability and unlikely redundancy in our study instrument.

In the overall cohort, 93.3% of the participants had low, 6.3% had moderate, and 0.8% had high willingness scores for assisting during an IME (Figure 1B). Similarly, 86.3% of the participants had low confidence, 12.2% had moderate confidence, and 1.5% had high confidence scores (Figure 1C). There were no differences in the proportion of participants with low, moderate, and high willingness or confidence scores by attendance in life support courses.

**Figure 2.**
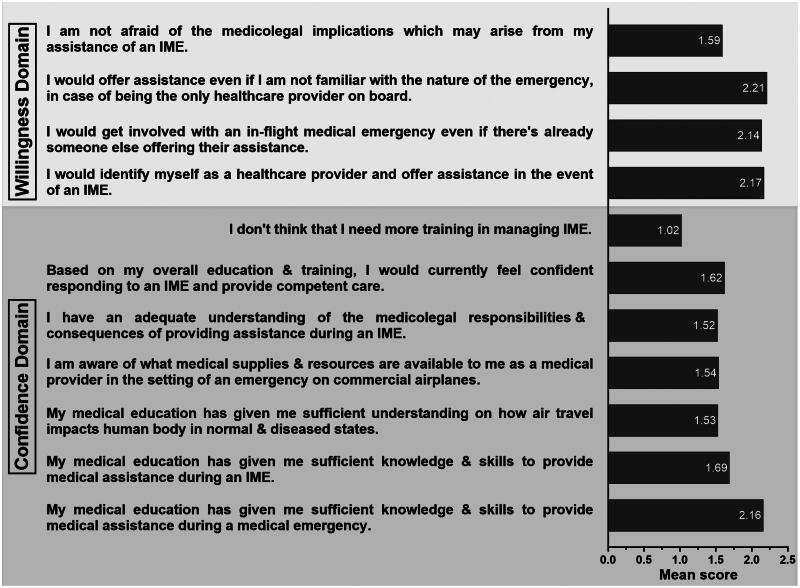
Mean Likert-scale scores for seven questions to test the participants’ confidence and four questions to test the participants’ willingness to provide medical assistance during an IME.

Only 40.9% of the participants ‘strongly agreed’ or ‘agreed’ that their medical education has prepared them to respond during an IME, while even fewer (24.4%) felt confident in responding to an IME. Similarly, only 38.3% of the participants stated they were willing to identify themselves and aid during an IME, and 49% were concerned about the medicolegal implications of providing care during an IME. Unsurprisingly, 72.1% of the participants ‘strongly disagreed’ or ‘disagreed’ that they did not need more training in managing IMEs. The mean Likert-scale scores for individual questions to test the confidence and willingness to provide medical assistance during an IME are presented in Figure 2.

## Discussion

This study demonstrates that medical students in Saudi Arabia had low confidence and willingness to provide care during an IME, which may be driven by their low IME-related knowledge. While the frequency of air travel ≥ two times per year was associated with higher odds of adequate knowledge score, there was no association between knowledge level and the type of medical school, GPA, a past encounter with an IME, attending life support courses, or completing an emergency medicine rotation, which may indicate a systemic defect. For example, the current Saudi medical school curriculum does not mandate the inclusion of air travel physiology, logistical considerations, clinical approach, or medicolegal implications related to managing IMEs. Although some students might pursue postgraduate training in emergency medicine, which has been previously shown to be associated with the highest scores of willingness and confidence in responding to IMEs, many of these students are likely to pursue other postgraduate career choices that might not adequately prepare them for such emergency scenarios [13,23].

Since 7 in 10 participants felt inadequately trained to assist during an IME, developing such training programs is urgently required. A few programs have recently been reported to train medical students to respond during emergencies. For instance, a simulation training program at the Department of Emergency Medicine, Christiana Care Health System, for third- and fourth-year medical students, which focused on onboard medical resources and patient management, improved knowledge scores for managing an IME [24]. The ‘First Five Minutes’ program at the Case Western Reserve University School of Medicine focused on scene safety, patient assessment, airway management, BLS/CPR, and hemorrhage control to train medical students as first responders during an emergency [25]. However, the ‘First Five Minutes’ program was not explicitly geared toward managing IMEs, and the program’s acceptability, feasibility, and efficacy have not yet been reported.

Relatively more IME training programs for emergency medicine residents have been described in the literature, which can be adapted to the needs and demands of senior medical students within the SaudiMED framework and the National Commission for Academic Accreditation and Assessment (NCAAA) that evaluates the quality of post-secondary programs [16]. For example, a hands-on simulation training program for emergency medicine residents at Vanderbilt University Medical Center focused on identifying roles and resources and patient evaluation and management during an IME [26]. The participants rated the program highly for improving their preparedness [26]. Another program at Ohio State University Wexner Medical Center conducted a realistic simulation training using a grounded Boeing 737 airliner at the local airport, which included five simulation-based learning (anaphylaxis, shockable rhythm arrest, syncope secondary to pulmonary embolism, anterior epistaxis, and an agitated passenger) and three discussion-based learning (aeromedical considerations, medicolegal topics, and decompression illness) for emergency and internal medicine residents [27]. The program improved participants’ medical knowledge, especially for ACLS, and self-assessed competency scores [27].

Additionally, app-based resources for decision-making during an IME have been described. In a randomized controlled trial, participants from non-emergency medicine residency programs with access to a mobile phone app (airRx) were likelier to complete the medical checklist [28]. However, during simulation sessions, timed activities such as cardiac and pulmonary exams or vitals assessments were significantly slower [28]. Slowness in medical students’ response to patients while using mobile device-based applications has also been noted in other medical domains, such as tobacco cessation counseling [29]. Popular medical podcasts, such as Curbsiders Internal Medicine Podcast, with an educational focus on IME management, are also available [30]. However, the clinical skills improvement among listeners of this program is yet to be reported.

Moreover, these training programs only briefly address, if at all, issues such as the medicolegal aspects of IME, which was also identified by over half of our study participants. IME presents unique medicolegal concerns for health professions students who wish to provide medical assistance. For example, jurisdiction is a common medicolegal concern as IME may occur over international waters or in airspace governed by different countries, creating jurisdictional issues for medical professionals who provide medical assistance. In addition, different laws exist to provide legal protection to volunteers providing reasonable assistance to strangers in the setting of an emergency. For example, in the United States, the United Kingdom, and Canada, physicians responding to an IME are generally protected under the Good Samaritan Law [31–33]. However, countries like France, Germany, and Denmark have implemented ‘duty to help’ legislation [34]. Therefore, volunteers may need to comply with different laws and regulations depending on the location of the aircraft and the nationality of the passengers and crew [35]. Unsurprisingly, physicians with a good understanding of these laws are more likely to intervene during an IME [31]. In Saudi Arabia, our literature review yielded no available guidelines or statements on IME by professional societies and associations.

Liability is yet another concern. Health professions students may be liable if they exceed their scope of practice or their actions harm the patient, which is an issue our research has identified, given that students were more willing to participate in IMEs despite their lack of adequate knowledge and confidence. While willingness to participate in IMEs can stem from good intentions, it can be dangerous, especially if a sole, inexperienced care provider feels compelled to act during an IME without understanding their role and limitations.

Along with medicolegal and liability topics, future training programs for IME will need to increase familiarity with the airline industry’s policies and procedures for handling medical emergencies and communicating with ground-based medical professionals [36,37]. Medical and other health professions students must also know the potential complications and limitations of providing medical care in an aircraft, such as limited space, equipment, and resources [9,38]. Moreover, in-flight medical emergencies may require health professions students to adapt their usual clinical practices to an aircraft’s unique environment and circumstances. For instance, they may need to prioritize interventions based on the severity of the patient’s condition and the available resources while considering the potential legal and ethical implications of providing care in a foreign jurisdiction [31,39]. Moreover, IMEs present a uniquely collaborative environment where health professionals must collaborate with other non-medically trained staff, such as the pilot, cabin crew, ground staff, and potentially fellow passengers. Therefore, medical students should be trained to work collaboratively to provide appropriate interventions and coordinate the transfer of care to the medical facility upon landing.

There are several ways in which medical and other health professions schools can ensure that their students receive adequate training on IME by incorporating the topic into the curriculum, either as a standalone module or as part of existing courses such as emergency medicine or critical care. High-fidelity simulations replicating the aircraft environment and scenarios for students to practice their skills and decision-making may be particularly effective [40]. Additionally, medical schools can encourage students to gain real-world experience by volunteering for medical assistance on flights or observing medical professionals specializing in aviation medicine. Simulations and case scenarios provide students with a unique opportunity to apply their knowledge and skills in a realistic setting, which can increase their self-efficacy and competence [41]. Such training may also help to reduce students’ anxiety and stress levels and increase their preparedness and willingness to intervene in such situations [42]. However, the already crowded medical curriculum and low topic priority may be barriers to incorporating IME training programs into regular medical education [43–45].

### Limitations

Our study has some limitations that restrict the generalizability of the findings. The convenience sample of participants in this study was more likely to be females and those attending public medical schools. There is a possibility of different outcomes with more diverse participant demographics. Further, using true/false questions in our questionnaire may introduce the possibility of random guessing among participants. This may impact the accuracy of our findings. While efforts were made to minimize this bias by providing clear instructions and emphasizing the importance of accurate responses, random guessing remains a limitation of our study.

However, the most significant limitation of our study is that the survey instrument was only assessed for face/content validity and internal consistency but not for construct and external validity. Also, as previously noted by other authors [14], there is a lack of externally validated questionnaires to investigate senior medical students’ knowledge, confidence, and willingness to respond to an IME. Therefore, acknowledging this limitation, we developed our conceptual framework and adapted the questions, drawing inspiration from previous research [13,14]. Our primary focus was selecting questions encompassing fundamental concepts pertinent to IMEs.

Creating a comprehensive set of externally validated questions is a highly intricate process exceeding the scope of this study as we aimed to provide the preliminary evidence that the current medical curriculum does not sufficiently train students to respond to an IME. It should be noted that only three studies have previously investigated medical students’ knowledge to respond to an IME, all indicating that medical students are ill-equipped to respond to an IME [14,24,40]. The confidence and willingness of medical students to respond to an IME has not been reported yet. Thus, the accumulating evidence from cross-sectional studies, including the current study and the recognition of the problem by experts from critical care, aviation medicine, and emergency medicine [46,47] as well as medical students [48], warrants the development of externally validated questionnaires to comprehensively investigate the knowledge, confidence, and willingness of senior medical students to respond to an IME. Given the international nature of air travel, such a survey instrument must be readily adaptable internationally, especially concerning geographical variations in medicolegal issues such as Good Samaritan laws. This would allow the comparison of student preparedness by country and ultimately aid in standardizing IME training programs globally.

Therefore, our findings should be interpreted cautiously and strictly hypothesis-generating until future research with validated survey instruments produces comparable results.

## Conclusion

Our results build on the limited number of previous studies, which confirm the presence of a gap in knowledge, confidence, and willingness to respond to IMEs among health profession students. Even though over 8 in 10 students in our study had previously attended life support courses, the overwhelming majority lacked the knowledge, confidence, and willingness to assist. Our study underscores the importance of teaching medical students about IMEs and their unique challenges before entering their 7th-year mandatory general internship.

In-flight medical emergencies can be complex and challenging to manage for medical students. Therefore, we recommend familiarizing students with the topic during medical school since many students might find themselves in a position as sole healthcare providers on an IME.

## Data Availability

The data supporting this study’s findings are available from the corresponding author, M.A, upon reasonable request.
